# A rare complication of a metacarpophalangeal joint replacement in a rheumatoid hand: a case report

**DOI:** 10.4076/1757-1626-2-7864

**Published:** 2009-09-10

**Authors:** Rajat Chopra, D K Jain, Raj Murali, E Gladston Chelliah

**Affiliations:** 1 Department of Clinical Research, Wrightington HospitalHall Lane, Appley Bridge, Wigan, Lancashire, WN6 9EPUK; 2 Department of Orthopaedics, Wrightington HospitalHall Lane, Appley Bridge, Wigan, Lancashire, WN6 9EPUK; 3 Department of Rheumatology, Wrightington HospitalHall Lane, Appley Bridge, Wigan, Lancashire, WN6 9EPUK

## Abstract

Metacarpophalangeal joint replacement is one of the most common surgery performed for rheumatoid hand deformities. The systemic and progressive nature of rheumatoid arthritis and other inflammatory arthritis make isolated assessment and treatment of metacarpophalangeal joint joints challenging.

Extensive joint involvement and systemic nature of the illness has an impact in the prognosis of the illness. The long term outcome of the surgical procedure depends on how best the illness is controlled. Technical aspects of the surgery in patients with rheumatoid arthritis can be widely variable and can make implant arthroplasty challenging. We present a case report of an unusual presentation of a rare complication following metacarpophalangeal joint replacement performed 17 years ago.

## Case presentation

**Figure 1.**
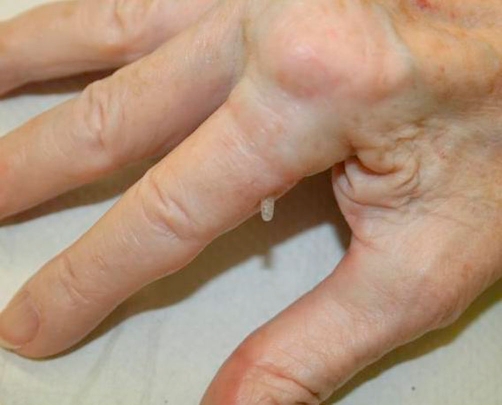
Protruded prosthesis coming out of the skin.

A 71-year-old British white woman presented to the combined rheumatology/orthopaedic clinic with a painful nodule over her replaced metacarpophalangeal joint (MCP) of her index finger (Figure 1). There were signs of skin inflammation around the nodule. She was seen earlier by her general practitioner and was prescribed oral antibiotics for a possible infected rheumatoid nodule. Skin inflammation responded to antibiotics but the nodule persisted. Later, she was referred to rheumatologist for their opinion.

On examination, a nodule was seen over the metacarpophalangeal joint of the index finger. There was a thin thread like material projecting from the nodule. The surrounding skin was normal. There was an old well-healed surgical scar over her MCP joint. The nodule was shiny and firm on palpation. It was not reducible. There was no movement at the level of metacarpophalangeal joint and little movement in the rest of joints of her finger. She did not have any blood investigations as the inflammation had settled after a course of oral antibiotics.

The clinical diagnosis of a possible peri-prosthetic fracture was made which was confirmed on radiological investigations. It was decided to explore and proceed with possible revision metacarpophalengeal joint replacement.

**Figure 2.**
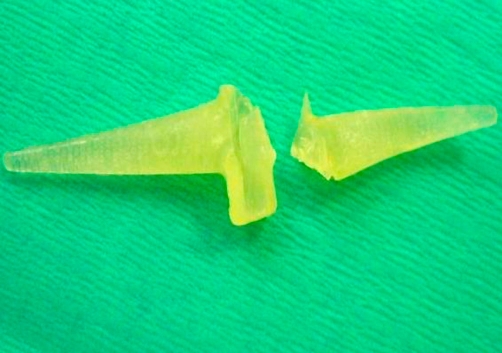
Removed silicone MCP prosthesis.

On exploration, the nodule on her finger was part of the prosthesis of metacarpophalangeal joint replacement as we previously thought (Figure 2). The prosthesis has gone through the bone and than through the skin. She underwent revision metacarpophalangeal joint replacement. Patient had satisfactory result of the revision joint replacement surgery.

## Discussion

MCP joint replacement in rheumatoid arthritis (RA) patients is one of the commonest surgeries performed for hand deformities and functional difficulties. Complications reported for a MCP joint replacement are namely implant fracture, periprosthetic cyst formation, subsidence, and recurrent deformity over time period [1-4]. Breakage rates have generally ranged between 0% and 30% [1,2,5,6], however, fracture rates as high as 82% at 5 years have been reported [1,7,8]. Other reported complications include delayed infection, silicone synovitis and lymphadenopathy, and rarely malignant lymphoma [9].

Protrusion of the prosthesis through the bone and than skin without any associated history of trauma is an extremely rare complication of metacarpophalangeal joint replacement. On reviewing literature, we found one case of index finger distal interphalangeal joint silicone arthroplasty, where the implant had eroded through the skin and was removed with satisfactory results [10].

Silicone arthroplasty for MCP joint replacement, introduced by Swanson in 1962, has remained the most popular procedure [5]. This is a constrained implant design and there are many constrained implants designs available in market. Long-term studies of these constrained implants demonstrate good pain relief, improved motion arc, correction of deformity, and high patient satisfaction [2,5,11]. While efforts are made to match the success of large total joint replacement, but difficulties are encountered when trying to transfer large joint technology to small joints of the hands. Most notable were the small size of joints, their place within the kinetic chain, complex soft tissue investments, and relationships to adjacent rays [12]. More recently, implants have moved toward semi-constrained or non-constrained designs and toward minimal bone resection that aims at preserving soft tissue supports to unload component stems and improve fixation while mimicking joint biomechanics. These implants are comparatively new in market and there long-term results are awaited.

## Conclusion

Our case is unique and very rare reported complication. It initially presented like an infected nodule that turned out to be a loose, extruded MCP joint replacement prosthesis. This presentation signifies that a careful history and examination is important in identifying rare clinical presentation like this.

## Abbreviations

MCP: metacarpophalangeal joint
RA: rheumatoid arthritis
